# Pesticide exposure affects reproductive capacity of common toads (*Bufo bufo*) in a viticultural landscape

**DOI:** 10.1007/s10646-020-02335-9

**Published:** 2021-01-20

**Authors:** Elena Adams, Christoph Leeb, Carsten A. Brühl

**Affiliations:** iES Landau, Institute for Environmental Sciences, University of Koblenz-Landau, Fortstraße 7, 76829 Landau, Germany

**Keywords:** Amphibians, Semi-field study, Fecundity, Population decline, Sublethal effects

## Abstract

Amphibian populations are declining worldwide at alarming rates. Among the large variety of contributing stressors, chemical pollutants like pesticides have been identified as a major factor for this decline. Besides direct effects on aquatic and terrestrial amphibian stages, sublethal effects like impairments in reproduction can affect a population. Therefore, we investigated the reproductive capacity of common toads (*Bufo bufo*) in the pesticide-intensive viticultural landscape of Palatinate in Southwest Germany along a pesticide gradient. In a semi-field study, we captured reproductively active common toad pairs of five breeding ponds with different pesticide contamination level and kept them in a net cage until spawning. Toads from more contaminated ponds showed an increased fecundity (more eggs) but decreased fertilization rates (fewer hatching tadpoles) as well as lower survival rates and reduced size in Gosner stage 25, suggesting that the higher exposed populations suffer from long-term reproductive impairments. In combination with acute toxicity effects, the detected sublethal effects, which are mostly not addressed in the ecological risk assessment of pesticides, pose a serious threat on amphibian populations in agricultural landscapes.

## Introduction

The latest IUCN reports suggest that 41% of all amphibian species are threatened (IUCN 2020). Besides habitat modification and destruction, intensive agriculture including the exposure to chemical pollutants like pesticides is one of the major factors for the global amphibian decline (Collins and Storfer 2003; Stuart et al. 2004). Several studies investigating the impact of intensive agriculture on amphibians determined adverse effects on egg and tadpole health (Babini et al. 2018), adult body condition, and morphology (Bionda et al. 2018; Hegde et al. 2019; Zhelev et al. 2017). One reason for these effects can be the exposure of amphibians to pesticides, with which they can come into contact during their whole life cycle. They can be exposed during the breeding phase and larval development in their aquatic habitats due to spray-drift (Crossland et al. 1982), run-off (Edwards et al. 1980) and drainages (Brown and van Beinum 2009). Post-metamorphic, terrestrial juvenile and adult amphibians can take up pesticides e.g., from contaminated soil (Storrs Méndez et al. 2009) during migration through the agricultural landscape (Leeb et al. 2020b; Lenhardt et al. 2013). Despite this chronic, biphasic exposure, the effects of chemical pollutants on amphibian declines is not well understood (Grant et al. 2016). Most ecotoxicological laboratory studies on amphibians focus on acute effects of pesticides that lead to direct mortality in aquatic or, more rarely studied, terrestrial life stages (e.g., Brühl et al. 2013; Relyea 2004, 2005). Besides these acute effects, chronic and sublethal effects due to impaired reproduction may also result in amphibian population declines. Thus, there is not only a potential for rapid but also long-term amphibian declines, either due to impairment of adult breeding or deficient development of a progeny (Hayes et al. 2010b).

On the one hand, sublethal effects on reproduction can occur due to direct systemic toxicity. Effects on molecular biomarkers like acetylcholine esterase activities (Hegde et al. 2019) and hematological parameters (Zhelev et al. 2018) as well as genotoxic and mutagenic effects (Gonçalves et al. 2019) may have an impact on the reproductive capacity and thus on amphibian populations. Moreover, resources for the production of eggs may be limited and reproduction reduced due to resources required for pesticide detoxification processes as shown for the woodlouse *Porcellio scaber* (Jones and Hopkin 1998). Pesticides may also indirectly affect amphibian reproduction by interfering with their food supply (Sánchez-Bayo and Wyckhuys 2019) or affecting their behavior and thus disturbing their habitat use (Leeb et al. 2020a), predation (Adams et al. 2020), mating behavior (Schwendiman and Propper 2012) and population connectivity (Lenhardt et al. 2017).

On the other hand, pesticides can also directly act on the hormonal pathways of developmental processes as endocrine disrupting chemicals (EDCs), which alter the normal functioning of the endocrine system leading to impaired reproduction mechanisms such as infertility or intersex (Ujhegyi and Bókony 2020). EDCs have been found in amphibian breeding sites in agricultural landscapes. Bókony et al. (2018) detected 41 EDCs across amphibian ponds in the agricultural landscape of Hungary. Müller and Zithier (2015) performed a monitoring of ten pesticides in small water bodies used by amphibians in agricultural landscapes in North Germany and detected amongst others the potential EDCs metazachlor and propiconazole. However, in general little information on pesticide contamination is available on water bodies used by amphibians for spawning and larval development, as most studies investigate pollution of groundwaters, river systems and lakes (Lorenz et al. 2017), neglecting small, shallow water bodies that are especially important for amphibians (Wells 2007).

Studies on direct reproduction effects of pesticides on amphibians are considerably rare. One of the few well-studied pesticides with endocrine disruptive properties is the insecticide atrazine that shows severe effects on the reproduction of amphibians. Larvae of African clawed frogs (*Xenopus laevis*) showed a decreased gonadal volume and germ cells (Tavera-Mendoza et al. 2002a, b) as well as a trend to hermaphroditism (Hayes et al. 2002b) after exposure to atrazine. Further, atrazine induced feminization of male leopard frogs (*Lithobates pipiens*) in nature (Hayes et al. 2002a). Pesticide mixtures containing atrazine also indirectly inhibit reproductive functioning, e.g., by increasing stress hormone levels like corticosterone in adult *X. laevis* (Hayes et al. 2006). This may lead to further impacts including inhibition of sex hormones (Burmeister et al. 2001) and the alteration of reproductive development, breeding behavior and fertility (Moore 1983). Other current-use pesticides with endocrine disruptive properties are for example dicarboxamides like the viticultural fungicide vinclozolin (Kortekamp et al. 2011). This fungicide has been shown to contribute to shifted sex ratios, an inhibited maturation and reduced fecundity as well as fertility in fish (Lor et al. 2015). Although a few studies have explored endocrine disrupting effects of viticultural azole fungicides like tebuconazole and penconazole (e.g., Lv et al. 2017; Poulsen et al. 2015), they are not yet considered as EDCs by the Pesticide Properties DataBase (PPBD, Agriculture and Environment Research Unit of the University of Hertfordshire 2013) and the PAN International List of Highly Hazardous Pesticides (PAN List of HHPs; Pesticide Action Network International 2019). Further pesticides may have similar effects, however, the database on endocrine disruptive properties is too small to allow for concrete conclusions.

Especially field data on sublethal reproduction endpoints are scarce because mainly laboratory studies are used to investigate effects of pesticides on reproduction. Thereby, the most investigated endpoint in field studies analyzing effects on reproduction is the incidence of intersex, in which individual´s gonads contain both female and male tissue (Ujhegyi and Bókony 2020). However, also other endpoints like the number of laid eggs, fertilization rates or the development success of early larvae can be used to evaluate effects of pesticides on the reproductive capacity. Bókony et al. (2018) investigated the effects of EDCs on common toads (*Bufo bufo*) in agricultural and urbanized ponds in Hungary and observed reduced developmental rates and lower body mass of the offspring compared to natural ponds.

Investigations on pesticide effects on the reproduction of amphibians in viticultural landscapes do not exist so far, although viticulture is one of the most pesticide-intensive cultures in Central Europe. On average 9.5 pesticide applications with a mixture of on average 1.6 formulations per application are performed during March and August in vineyards (Roßberg 2009). Because of the combined aquatic and terrestrial exposure of amphibians to viticultural pesticides, long-term adverse effects on reproduction are likely. To address this lack of knowledge, we investigated the reproductive capacity of common toads (*Bufo bufo*, LINNAEUS 1758) in the viticultural landscape of Palatinate in Southwest Germany along a pesticide gradient. We hypothesized that an increased chronic pesticide exposure affects fecundity, fertilization rate as well as offspring survival and size. Common toads were used since it is the most common amphibian species in Central Europe (Sillero et al. 2014) and it occupies a broad range of habitat types including agricultural landscapes like vineyards (Leeb et al. 2020b; Lenhardt et al. 2013). They are not yet considered endangered on an international as well as national level (Agasyan et al. 2009; Kühnel et al. 2009). However, population declines have been observed on a local level (e.g., Beebee and Griffiths 2005; Bonardi et al. 2011; Kyek et al. 2017; Petrovan and Schmidt 2016).

## Material and methods

### Study sites and exposure assessment

**Fig. 1:**
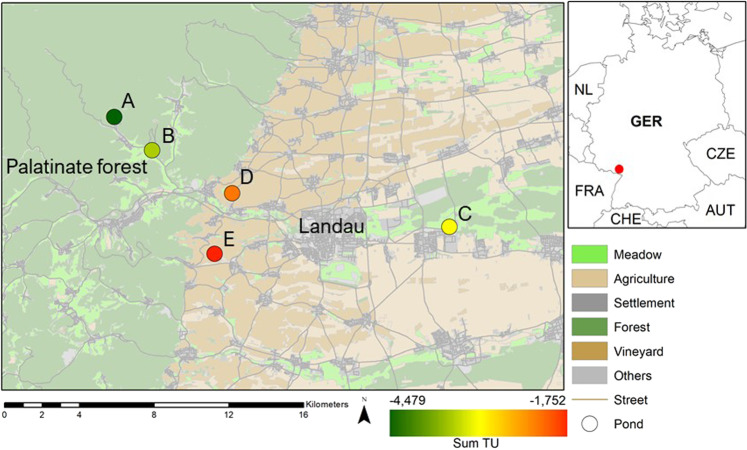
Map of study ponds in Palatinate in Southwest Germany. Increasing letters and colors of study sites represent the pesticide contamination from no contamination (dark-green, Pond A) to high contamination (red, Pond E). Source: Basemap: DLM50—^©^GeoBasis-DE/LVermGeoRP2020, dl-de/by-2-0, www.lvermgeo.rlp.de [modified data]

In spring 2019, we studied common toad populations from five ponds (pond A–E, Table 1, Fig. 1) around Landau, one of the largest winegrowing areas in Southwest Germany. These ponds were expected to represent a gradient of pesticide contamination due to their varying agricultural surrounding. For validation of the pesticide gradient, five water samples were collected of each pond between April and May 2018 and analyzed for 47 different fungicides, six insecticides, three herbicides, and two acaricides (Table S1) by the Institute of Phytomedicine of the Dienstleistungszentrum Ländlicher Raum Rheinpfalz in Neustadt/Weinstraße, Germany. The selection of analyzed pesticides was based on spraying recommendations for vine from local authorities (www.dlr.rlp.de).

**Table 1:** Locations of study ponds, contamination level (sum of toxic units, STU, see Eq. 2), number of captured toad pairs and number of toad pairs that spawned

Pond	Coordinates (WGS84)	STU	Number of toad pairs	
			Captured	Spawned
A	49.25475, 7.96182	−4.48	12	11
B	49.23830, 7.99002	−3.48	13	11
C	49.20329, 8.20917	−3.09	15	13
D	49.21830, 8.04944	−2.25	14	14
E	49.18898, 8.03709	−1.75	8	5

Pond letters indicate increasing STU. Since no pesticides were detected in pond A, its STU was calculated based on the use of 1/10 of the minimum TU observed in the sites with detected concentrations (for rationale s. Schäfer et al. 2011)

The pond pesticide toxicity was assessed using Toxic Units for each detected pesticide (Eq. 1, with *C*_i_ = detected concentration of pesticide *i* and LC50_*i*_ = median lethal concentration causing 50% mortality of test organisms).

1 $$TU \,=\, \frac{{C_i}}{{LC50_i}}.$$

As LC50 values for amphibians are often lacking, data of acute fish toxicity studies compiled from the PPDB (Agriculture and Environment Research Unit of the University of Hertfordshire 2013) were used as proxy for amphibians (Weltje et al. 2013). The sum of TU (STU, Eq. 2, with *n* = number of detected pesticides) was calculated to aggregate the toxicity of the detected pesticides (Table 1, Schäfer et al. 2011) by using the maximum detected sum of TU of each study pond. To allow the comparison to sites without any detected pesticides, uncontaminated ponds were assigned to a TU of 1/10 of the minimum TU observed in the contaminated sites (Fernández et al. 2015), leading to a STU of −4.48 for pond A.

2 $$STU \,=\, {\text{log}}\left( {{\text{max}}\mathop {\sum }\limits_{i \,=\, 1}^n TU} \right).$$

The detected pesticides were checked for endocrine disruptive properties using toxicity data from the PPDB (Agriculture and Environment Research Unit of the University of Hertfordshire 2013) and the PAN List of HHPs (Pesticide Action Network International 2019). Moreover, acute and chronic regulatory acceptable concentrations (RACs) were calculated based on fish toxicity values from the PPDB (LC50 and NOEC = No observed effect concentration, Eqs. 3, 4, Table S2). As uncertainty factors, 100 was used for the acute and 10 for the chronic RAC as recommended for aquatic organisms by EFSA (2013). The RACs were compared to the detected concentrations to estimate the acute and chronic aquatic toxicity of the ponds.

3 $$RAC_{acute} \,=\, \frac{{LC50}}{{100}}.$$

4 $$RAC_{chronic} \,=\, \frac{{NOEC}}{{10}}.$$

Moreover, the landscape composition around the study ponds was analyzed. Based on a vector landscape model of Rhineland-Palatinate (ATKIS DLM50), the percentages of vineyards, other agriculture, meadows, settlements, and forests were calculated. A radius of three kilometer was chosen to analyze the landscape composition because this distance reflects the annual migrations between hibernation as well as summer habitats and breeding ponds for *B. bufo* (Günther 2009). To estimate the terrestrial exposure, data of viticultural and other agricultural area was used.

### Reproductive capacity analysis

We aimed to capture ten or more reproductively active adult common toad pairs during their spawning season between 9 and 28 March 2019 from each pond. After capturing, each pair was housed in a net cage (80 × 65 × 60 cm) in the respective breeding pond containing a wire hanger as spawning substrate. Due to the short spawning season of *B. bufo* and the fact that not all pairs spawned, it was not possible to investigate ten spawning pairs of each pond (Table 1). Finally, we captured 62 toad pairs from which eight pairs did not spawn, 45 pairs spawned within 7 days and nine pairs within 15 days after catchment. One day after spawning, the body mass of each toad was measured (±0.1 g) and the individuals were released in their ponds. It can be assumed that females laid all eggs at once because the spawning process is usually finished after 6 to 12 h (Günther 2009) and the pairs terminated the amplexus after oviposition.

As measures of each population’s reproductive capacity, we analyzed the fecundity, fertilization rate, offspring survival until the free-swimming Gosner Stage 25 (GS; Gosner 1960) and offspring size (tadpole length) at GS25. To determine the fecundity, the number of laid eggs per female was counted. Because fecundity is known to increase with female size (Banks and Beebee 1986; Reading 1986), we calculated the ratio of the amount of laid eggs and the body mass of the females after spawning (eggs/g body mass). To estimate the fertilization rate and offspring survival, approximately 90 eggs of each clutch were removed from three randomly chosen parts of the egg string and kept individually in clear plastic aquariums (22.5 × 16.5 × 7 cm, Braplast, Bergheim, Germany) filled with 1 L FETAX medium (Dawson and Bantle 1987). To prevent any injuries of eggs, the handling of the spawning strings was kept to a minimum. Thus, the number of eggs was not identical for each sample. Because mold grew on the first three egg strings collected from pond C, three samples of pond C could not be used to analyze the fertilization rate and offspring survival. To prevent mold from growing on further eggs, eggs of one egg string were separated but still incubated together in one aquarium. The eggs were reared in a climate chamber at 21 ± 1 °C and a 16:8 h day:night light cycle until they reached GS25. The individuals were photographed daily. Three days after spawning, non-fertilized eggs that exhibited mold growing on them or did not show embryonic development were removed. Developing eggs were counted using Image J (Schneider et al. 2012) to calculate the fertilization rate. Fertilized eggs from one egg string hatched within a time difference of maximum 24 h. As soon as all tadpoles reached GS25 (9–10 days), the proportion of embryos that survived to this stage was counted to estimate the offspring survival. Moreover, the lengths of twelve randomly selected tadpoles of each sample were determined to estimate the offspring’s sizes. After recording the needed data, the tadpoles were released in their origin pond.

### Statistical analyses

Statistical analyses were performed using R (version 3.5.2; R Core Team 2013). To determine the correlation of the aquatic and terrestrial exposure, a Pearson´s correlation was performed. Kendall-Theil Sen Siegel non-parametric regressions (Sen 1968; Siegel 1982; Theil 1950) were performed to check whether the investigated endpoints depend on the pesticide contamination of ponds (STU). Moreover, Spearman’s rank correlations between the investigated endpoints and the STU of ponds were computed (Spearman’s rank correlation coefficient *ρ*, Hollander et al. 1973).

To check the assumption that fecundity is increased by female size, a Spearman’s rank correlation was performed for the female body mass and the number of laid eggs. Moreover, Spearman’s rank correlations were performed to investigate the relationship between the pesticide contamination (STU) and the female body mass, the number of laid eggs and the tadpole length in GS25, parental body masses and the fertilization rate as well as the number of laid eggs per female and the fertilization rate. To investigate a measure of population fitness, the product of the four investigated reproductive endpoints was calculated and a one-way analysis of variance (ANOVA) was performed to identify differences between the investigated ponds. Tukey’s method was used to identify and remove outliers ranged above and below the 1.5 × IQR (Kannan Senthamarai et al. 2015). For all statistical tests, the criterion for significance was set to *α* = 0.05.

## Results

### Exposure assessment

The pesticide residue analysis revealed 22 different pesticides in total and 0–19 different pesticides per pond with a STU between −4.48 and −1.75 (Tables 1, S2) meaning no aquatic toxicity at a STU of −4.48 and high toxicity at a STU of −1.75. Toxicity data extracted from the PPDB and the PAN List of HHPs for the detected pesticides did not show any endocrine disruptive properties or the data base was insufficient to make a statement about endocrine disruptive properties. However, azole fungicides which were shown to be potential EDCs (Kortekamp et al. 2011; Lv et al. 2017; Poulsen et al. 2015) were detected in the ponds. Penconazole was detected in ponds B, D and E (0.02–0.18 µg/L), tebuconazole in ponds C, D and E (0.05–0.08 µg/L) and difenconazole in pond C (0.02 µg/L).

The comparison of detected concentrations to RACs revealed a conspicuous toxicity of the chronic exposure to the fungicides folpet and famoxadone and the acute exposure to famoxadone in pond E (Table S2). The chronic RAC of folpet was 5.6 times lower than the detected concentration in sampling 2 (4.53 µg/L), the chronic RAC of famoxadone was 1.1 times lower and the acute RAC of famoxadone was 1.4 times lower than the detected concentration in sampling 5 (0.15 µg/L), resulting in an increased hazard of adverse effects.

The landscape composition analysis showed an increasing agricultural land-use from pond A to pond E in a three-kilometer radius around the study ponds ranging from 0 to 60% (Table 2). The Pearson correlation revealed a statistically significant correlation between the STU and the agricultural land-use (*p* = 0.02, Pearson’s *r* = 0.94, df = 3).

**Table 2:** Landscape composition in a radius of 3000 m around the study ponds based on a vector landscape model of Rhineland-Palatinate (ATKIS DLM50)

Pond	Viticulture [%]	Other agriculture [%]	Meadow [%]	Settlement [%]	Forest [%]	Other [%]
A	0.0	0.0	5.1	1.3	92.9	0.6
B	0.1	1.1	19.2	5.6	72.1	1.9
C	0.3	31.4	19.6	15.5	28.5	4.8
D	47.5	1.1	7.9	11.6	29.8	2.2
E	57.0	3.1	6.1	10.1	22.5	1.3

### Reproductive capacity

Neither the female body mass (52.0 ± 14.1 g), the male body mass (33.46 ± 6.7 g), nor the number of laid eggs per female (3243 ± 1538) affected the fertilization rate (*ρ* = −0.24, *p* = 0.10, *ρ* = −0.09, *p* = 0.56 and *ρ* = −24, *p* = 0.10). The female body mass was positively correlated with the number of laid eggs (*ρ* = 0.62, *p* < 0.001) and the STU (*ρ* = 0.38, *p* < 0.01). Moreover, the offspring size (tadpole length in GS25) was negatively correlated with the number of laid eggs per female (*ρ* = −0.32, *p* = 0.03).

**Fig. 2:**
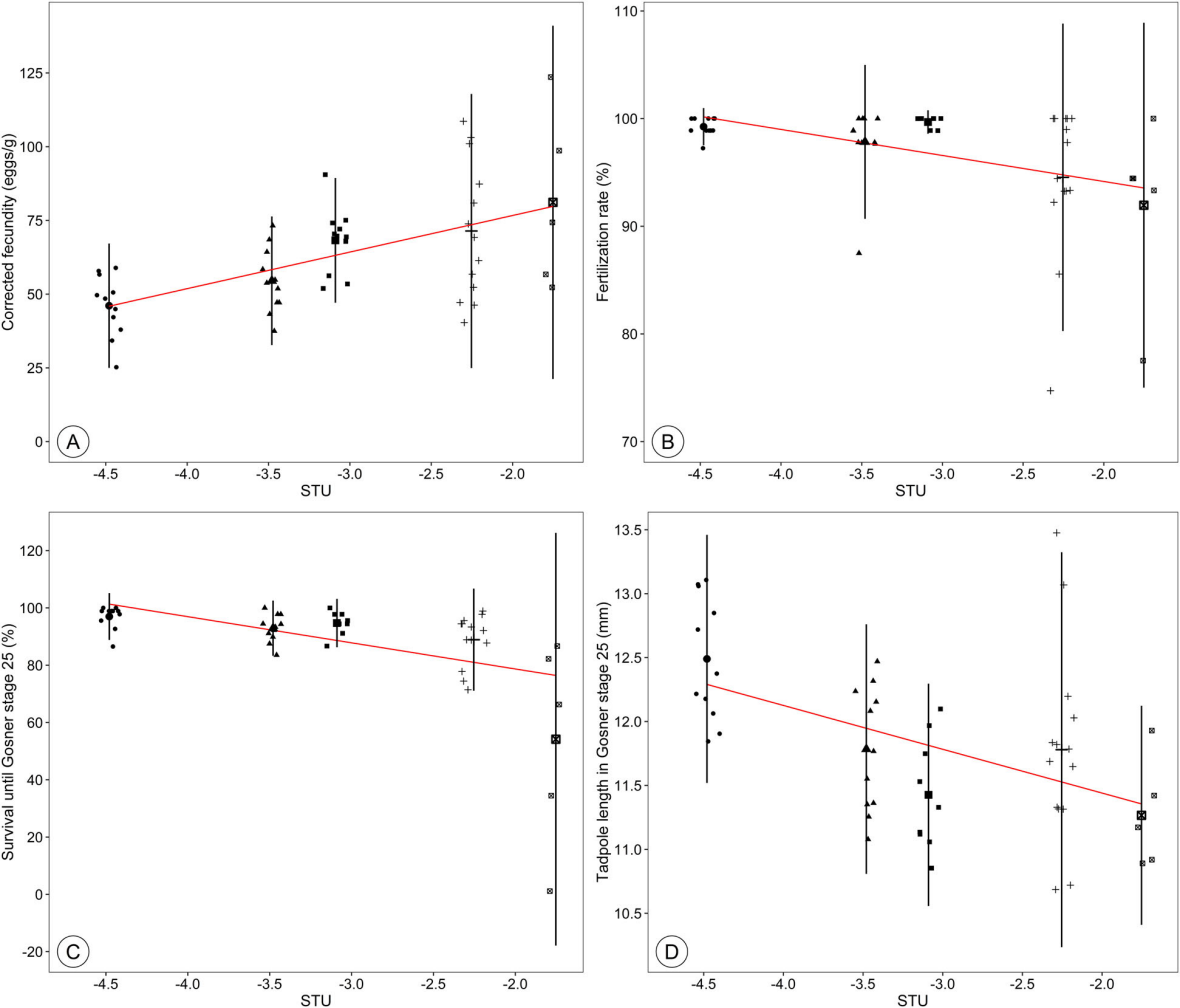
Dependence of fecundity (**A**), fertilization rate (**B**), offspring survival until Gosner stage 25 (**C**) and offspring size in Gosner stage 25 (**D**) on the pesticide contamination of breeding ponds (maximum sum of toxic units, STU). Fecundity was corrected for the body mass of the females after spawning (eggs/g body mass). For each pond, the means and standard deviations are presented (Table S4)

Kendall-Theil Sen Siegel regressions revealed a significant influence of the STU on all investigated endpoints (*p* < 0.001, Table S3). The mean fecundity differed from 49 to 74 eggs/g body mass and showed a positive correlation with increasing STU (*ρ* = 0.54, *p* < 0.001, Fig. 2A, Table S4). The fertilization rate, offspring survival and tadpole lengths showed mean decreases of 4.5%, 32.6% and 10.7% with increasing STU (Fig. 2A–D, Table S4). Negative correlations between the STU and the fertilization rate (*ρ* = −0.32, *p* = 0.03, Fig. 2B), the offspring survival (*ρ* = −0.57, *p* < 0.001, Fig. 2C) as well as the offspring size (*ρ* = −0.49, *p* < 0.001, Fig. 2D) were observed. The performed ANOVA did not reveal any differences for population fitness between the study ponds (*p* > 0.05).

## Discussion

### Exposure assessment

Since pesticide contamination of ponds are often reported to correlate with the surrounding agricultural land-use (Baker 2006), it was assumed that the detected pesticide gradient also represents the exposure during the pre- and post-breeding migration of the terrestrial amphibian stages. The determined correlation of aquatic exposure and land-use confirms this hypothesis.

No general statement can be drawn about the endocrine disruptive potential of the detected pesticides because further research is needed on their potential to act as EDCs. The well-studied endocrine disrupting herbicide atrazine was not detected in any of the study ponds probably because it is prohibited in Germany since 1991. However, since potentially endocrine disruptive pesticides like the azole fungicides penconazole, tebuconazole and difenconazole were detected, similar endocrine effects are likely. Furthermore, the ponds were only analyzed for active ingredients of pesticides. A statement about the toxicity of product additives, which can have a high acute toxicity, endocrine disruptive or reproductive toxic properties themselves or as metabolite (Mesnage and Antoniou 2017; Mullin et al. 2016), cannot be made.

The comparison of detected concentrations to chronic RACs of folpet and famoxadone in pond E reveals a high toxicity for aquatic vertebrates. Next to possible adverse effects because of single pesticides, mixture effects in ponds with up to 19 detected pesticides may contribute to higher toxicities (Relyea 2009). Moreover, it cannot be excluded that even higher concentrations and further pesticides were present in the ponds due to the limited number of water samplings (*n* = 5) and analyzed pesticides (*n* = 58 target molecules). Since only one rain event sampling was performed in the present study, peak pesticide concentrations may be underestimated (Neumann et al. 2003). Especially folpet and famoxadone may be present at higher concentrations than detected because they have very short dissipation times in water (DT50 folpet = 0.02 d, DT50 famoxadone = 0.1 d, Agriculture and Environment Research Unit of the University of Hertfordshire 2013).

### Reproductive capacity

Toads of the highest contaminated pond E showed on average a 1.5 times higher fecundity than toads of the uncontaminated pond A. In comparison to the present study, Bókony et al. (2018) did not observe any effect on the fecundity of common toads in agricultural ponds compared to natural ponds. Because the female body mass correlated with the number of eggs and both of them correlated with STU, the increased fecundity may be based on the higher female body masses in the contaminated ponds. Guillot et al. (2016) also observed larger and heavier common toads in French agricultural habitats compared to uncontaminated forest habitats. The increased body sizes might either suggest a potential adjustment during aging or some habitat specificities in the agricultural landscape may enhance body size. For example, smaller population densities in agricultural landscapes might decrease intra- and/or interspecific competition leading to larger individuals (Bishop et al. 1999; Guillot et al. 2016; Janin et al. 2011). However, there are multiple reasons that may affect adult body size without an agricultural context such as climate, habitat geography, size at metamorphosis, and availability of food resources.

The fertilization rate was negatively affected with increasing pesticide contamination of the ponds, suggesting that the higher exposed populations suffer from long-term reproductive impairments. There are several reasons that may have led to the observed decreased fertilization rate. Due to the increased number of eggs per female, the male fertilization success may be reduced. But also behavioral impairments during mating could lead to decreased fertilization rates. Hayes et al. (2010a) observed a reduced success of amplexus in male *X. laevis* exposed to atrazine and thus a lower proportion of fertilized eggs for atrazine exposed males. Also endocrine disruptive properties of pesticides may have led to this decrease for example due to impaired spermatogenesis which already has been reported after the exposure of frogs to the herbicide atrazine. Hayes et al. (2010a) observed a decreased frequency of testicular tubules with mature spermatozoa in *X. laevis*. In *X. laevis* tadpoles a reduction in testicular volume during sexual differentiation of the testis was observed (Tavera-Mendoza et al. 2002b). Another reason may be an effect on female sexual development. In-vitro assays with eleven pesticides of Orton et al. (2009) revealed altered ovarian steroidogenesis and reduced progesterone production. Pickford and Morris (2003) investigated the effects of the insecticide methoxychlor on female *X. laevis* and detected an inhibition of oviposition and maturation of oocytes. Moreover, the exposure to atrazine caused a reduction in the number of germ cells in the ovary and an increase of damaged oocytes (Tavera-Mendoza et al. 2002b). The larval exposure of *X. laevis* to atrazine induced a reduction of testosterone levels in males (Hayes et al. 2010a) leading to a decrease of male reproductive success (Moore and Hopkins 2009).

Decreasing survival rates and tadpole sizes were observed with increasing pesticide contamination. Bókony et al. (2018) also observed reduced body masses of common toad larvae and juveniles in agricultural landscapes in comparison to natural landscapes. Clearly, decreased survival of the tadpoles directly leads to population declines. The reduced tadpole lengths could lead to further impairments since body size is a critical determinant of individual fitness (Wells 2007). Smaller tadpoles sizes lead to reduced sizes at metamorphosis and thus to a decreased survivorship of the first hibernation (Üveges et al. 2016) and until maturity as well as delayed achievement of reproductive size (Smith 1987). Reduced body size is also a disadvantage as adult for reproduction because it affects female fecundity and male mating success (Banks and Beebee 1986; Davies and Halliday 1979; Reading et al. 1991).

On the one hand, reduced offspring size may be a long-term consequence of chronic pesticide pollution over several generations. Transgenerational effects were observed in rats after the exposure to EDCs as Anway et al. (2005) detected a decreased spermatogenic capacity in cell number and viability as well as an increase of male infertility in four tested generations. Thus, early-life exposure of parents can lead to impaired offspring viability. To verify the proposed reasons of reproduction impairments regarding endocrine disruptive effects, tissue analyses of e. g. thyroids and gonads would be needed. However, the present study was designed and completed without any lethal interferences and tissue withdrawals of the amphibian populations.

On the other hand, the reduced offspring size originating from highly contaminated ponds may be a cost of an evolutionary adaptive resistance (Whitehead et al. 2012) or of detoxification processes of contaminants (Rix et al. 2016). Similar effects have been observed for urban fish populations which evolved tolerance to toxic pollutants (Meyer and Di Giulio 2003; Whitehead et al. 2012). However, their offspring showed reduced growth rates and were more susceptible to other stressors compared with the offspring from a non-contaminated site (Meyer and Di Giulio 2003). Similar trade-offs may be responsible for the smaller tadpoles of the more contaminated ponds. Adult toads of these ponds may invest more resources into the production of egg jelly coat material to provide a better protection against pesticides. These resources may have in turn not be invested into larger ova (Podolsky 2004) which may have led to smaller tadpoles such as determined by Kaplan (1980). The higher egg production in contaminated ponds may be discussed as an adaptation to increase fitness by counterbalancing negative pesticide effects on embryo and tadpole development by an increased egg number.

Although amphibians are especially affected by pesticides due to their biphasic lifecycle, they are not yet considered in the environmental risk assessment of pesticides in the EU (Ockleford et al. 2018). Our data support the suggestion of inhibitory effects of current-use pesticides on the reproductive capacity of amphibians, potentially contributing to population declines. Thus, not only acute effects should be investigated in ecotoxicological amphibian studies but also sublethal effects on reproduction on a population level. Since data involving field scenarios analyzing the effects of multiple pesticides on amphibian reproduction are considerably rare, our results are of significant importance for amphibian conservation in agricultural landscapes.

## Supplementary information

Table S1

Table S2

Table S3

Table S4

## Data Availability

Data are available by contacting EA (adams@uni-landau.de).

## Code availability

The used R code is available by contacting EA (adams@uni-landau.de).
