# Supplementary material for: Pesticide exposure affects reproductive capacity of common toads (Bufo bufo) in a viticultural landscape
Source: Ecotoxicology. 2021 Jan 20;30(2):213–23. doi: 10.1007/s10646-020-02335-9 (PMC7902574; doi:10.1007/s10646-020-02335-9)
Supplement: Supplementary file 1 — Table S1 [file 10646_2020_2335_MOESM1_ESM.pdf]

Supplementary material Table S1

Pesticide exposure affects reproductive capacity of common toads (*Bufo bufo*) in a viticultural landscape

Elena Adams[1*], Christoph Leeb[1], Carsten A. Brühl[1]
[1]iES Landau, Institute for Environmental Sciences, University of Koblenz-Landau, Fortstraße 7, 76829 Landau, Germany
*Corresponding author: adams@uni-landau.de

Table S1. Investigated target pesticides of the aquatic residual analysis. In total 47 different fungicides, six insecticides, three herbicides and two acaricides were investigated.

Fungicides	Insecticides	Herbicides	Acaricides
Amisulbrom	Chlorpyrifos-methyl	Atrazine	Spirodiclofen
Azoxystrobin	Dimethoate	Carfentrazone-ethyl	Tebufenpyrad
Benalaxyl-M	Indoxacarb	Simazine	
Benthivalicarb	Methidathion		
Boscalid	Parathion-ethyl		
Captan	Parathion-methyl		
Cyazofamid			
Cyflufenamid			
Cyprodinil			
Dichlofluanid			
Difenoconazole			
Dimethomorph			
Epoxiconazole			
Famoxadone			
Fenarimol			
Fenhexamid			
Fenpropimorph			
Fenpyrazamine			
Folpet			
Fludioxonil			
Fluopicolide			
Fluopyram			
Fluquinconazole			
Iprodion			
Iprovalicarb			
Kresoxim-methyl			
Metalaxyl M			
Metrafenone			
Myclobutanil			
Penconazole			
Prochloraz			
Procymidon			
Propinconazole			
Proquinazid			
Pyraclostrobin			
Pyrifenox			
Pyrimethanil			
Quinoxyfen			
Spiroxamin			
Tebuconazole			
Tetraconazole			
Tolylfluanid			
Triadimefon			
Triadimenol			
Trifloxystrobin			
Vinclozolin			
Zoxamide			
