# Supplementary material for: Pesticide exposure affects reproductive capacity of common toads (Bufo bufo) in a viticultural landscape
Source: Ecotoxicology. 2021 Jan 20;30(2):213–23. doi: 10.1007/s10646-020-02335-9 (PMC7902574; doi:10.1007/s10646-020-02335-9)
Supplement: Supplementary file 2 — Table S2 [file 10646_2020_2335_MOESM2_ESM.pdf]

Supplementary material Table S2

Pesticide exposure affects reproductive capacity of common toads (*Bufo bufo*) in a viticultural landscape

Elena Adams[1*], Christoph Leeb[1], Carsten A. Brühl[1]
[1]iES Landau, Institute for Environmental Sciences, University of Koblenz-Landau, Fortstraße 7, 76829 Landau, Germany
*Corresponding author: adams@uni-landau.de

Table S2. Detected pesticide concentrations in the aquatic residual analysis of each study pond and respective risk assessment parameters. In total five samplings were performed (06.04.2018, 19.04.2018, 03.05.2018, 10.05.2018 - rain event, 24.05.2018). Pond letters indicate increasing pesticide contamination (based on the sum of toxic units).

DC = Detected concentration; NOEC = No observed effect concentration (chronic toxicity) for fish and LC50 = Median lethal concentration causing 50% mortality of fish (acute toxicity) were extracted from the Pesticide Properties Database (Agriculture and Environment Unit of the University of Herfortshire 2013); NOEC-RAC = Regulatory acceptable concentration based on the NOEC values, which are divided by the assessment factor for chronic studies (10); LC50-RAC = Regulatory acceptable concentration based on the LC50 values, which are divided by the assessment factor for acute studies (100). Detected concentrations were divided by calculated RAC values. A result > 1 (highlighted in bold) reveals a possible hazard for aquatic organisms.

Sampling	Pesticide	DC (µg/L)	NOEC (µg/L)	NOEC-RAC (µg/L)	DC/NOEC-RAC	LC50 (µg/L)	LC50-RAC (µg/L)	DC/LC50-RAC
**Pond A**								
1-5	-	-	-	-	-	-	-	-
**Pond B**								
1	Boscalid	0.09	125	12.5	0.0072	2700	27	0.0033
	Fludioxonil	0.04	40	4	0.0100	230	2.3	0.0174
	Dimethoate	0.03	400	40	0.0008	30200	302	0.0001
2	Boscalid	0.02	125	12.5	0.0016	2700	27	0.0007
	Zoxamide	0.03	4	0.4	0.0750	160	1.6	0.0188
3	Dimethoate	0.03	400	40	0.0008	30200	302	0.0001
4	Fludioxonil	0.06	40	4	0.0150	230	0.23	0.26
	Myclobutanil	0.14	200	20	0.0070	2000	2	0.07

	Dimethoate	0.02	400	40	0.0005	30200	30.2	0.0007
5	Boscalid	0.02	125	12.5	0.0016	2700	27	0.0007
	Fludioxonil	0.07	40	4	0.0175	230	2.3	0.0304
	Penconazole	0.02	360	36	0.0006	1130	11.3	0.0018
**Pond C**								
1	Azoxystrobin	0.04	147	14.7	0.0027	470	4.7	0.0085
	Difenconazole	0.02	23	2.3	0.0087	1100	11	0.0018
	Fludioxonil	0.07	40	4	0.0175	230	2.3	0.0304
	Iprovalicarb	0.28	9890	989	0.0003	22700	227	0.0012
	Metalaxyl M	0.08	9100	910	0.0001	27000	270	0.0003
	Myclobutanil	0.03	200	20	0.0015	2000	20	0.0015
	Dimethoate	0.05	400	40	0.0013	30200	302	0.0002
2	Azoxystrobin	0.03	147	14.7	0.0020	470	4.7	0.0064
	Fluopyram	0.02	135	13.5	0.0015	42900	429	0.0000
	Zoxamide	0.12	4	0.4	0.3000	160	1.6	0.0750
3	Folpet	0.33	8.1	0.81	0.4074	680	6.8	0.0485
	Iprovalicarb	0.09	9890	989	0.0001	22700	227	0.0004
4	Azoxystrobin	0.02	147	14.7	0.0014	470	4.7	0.0043
	Fludioxonil	0.05	40	4	0.0125	230	2.3	0.0217
	Iprovalicarb	0.11	9890	989	0.0001	22700	227	0.0005
	Myclobutanil	0.04	200	20	0.0020	2000	20	0.0020
	Pyrimethanil	0.02	1600	160	0.0001	10560	105.6	0.0002
	Dimethoate	0.03	400	40	0.0008	30200	302	0.0001
5	Cyflufenamid	0.02	24	2.4	0.0083	1040	10.4	0.0019
	Folpet	0.22	8.1	0.81	0.2716	680	6.8	0.0324
	Fludioxonil	0.07	40	4	0.0175	230	2.3	0.0304
	Iprovalicarb	0.11	9890	989	0.0001	22700	227	0.0005
	Metrafenone	0.10	228	22.8	0.0044	820	8.2	0.0122
	Myclobutanil	0.10	200	20	0.0050	2000	20	0.0050
	Tebuconazole	0.07	10	1	0.0700	4400	44	0.0016
	Chlorpyrifos-methyl	0.02	5	0.5	0.0400	410	4.1	0.0049

	Dimethoate	0.04	400	40	0.0010	30200	302	0.0001
**Pond D**								
1	Dimethomorph	0.06	56	5.6	0.0107	6100	61	0.0010
	Famoxadone	0.06	1.4	0.14	0.4286	11	0.11	0.5455
	Iprovalicarb	0.44	9890	989	0.0004	22700	227	0.0019
	Metalaxyl M	0.08	9100	910	0.0001	27000	270	0.0003
	Myclobutanil	0.22	200	20	0.0110	2000	20	0.0110
	Dimethoate	0.03	400	40	0.0008	30200	302	0.0001
2	Boscalid	0.04	125	12.5	0.0032	2700	27	0.0015
	Fluopicolide	0.04	155	15.5	0.0026	360	3.6	0.0111
	Zoxamide	0.04	4	0.4	0.1000	160	1.6	0.0250
3	Dimethoate	0.03	400	40	0.0008	30200	302	0.0001
4	Boscalid	0.02	125	12.5	0.0016	2700	27	0.0007
	Cyflufenamid	0.02	24	2.4	0.0083	1040	10.4	0.0019
	Fludioxonil	0.19	40	4	0.0475	230	2.3	0.0826
	Fluopyram	0.17	135	13.5	0.0126	42900	429	0.0004
	Iprovalicarb	0.12	9890	989	0.0001	22700	227	0.0005
	Myclobutanil	0.06	200	20	0.0030	2000	20	0.0030
	Penconazole	0.18	360	36	0.0050	1130	11.3	0.0159
	Pyrimethanil	0.04	1600	160	0.0003	10560	105.6	0.0004
	Tebuconazole	0.08	10	1	0.0800	4400	44	0.0018
	Chlorpyrifos-methyl	0.02	5	0.5	0.0400	410	4.1	0.0049
	Dimethoate	0.08	400	40	0.0020	30200	302	0.0003
5	Famoxadone	0.05	1.4	0.14	0.3571	11	0.11	0.4545
	Fludioxonil	0.06	40	4	0.0150	230	2.3	0.0261
	Iprovalicarb	0.11	9890	989	0.0001	22700	227	0.0005
	Myclobutanil	0.03	200	20	0.0015	2000	20	0.0015
	Dimethoate	0.03	400	40	0.0008	30200	302	0.0001
**Pond E**								
1	Azoxystrobin	0.07	147	14.7	0.0048	470	4.7	0.0149
	Boscalid	0.27	125	12.5	0.0216	2700	27	0.0100

	Fludioxonil	0.07	40	4	0.0175	230	2.3	0.0304
	Fluopicolide	0.04	155	15.5	0.0026	360	3.6	0.0111
	Fluopyram	0.21	135	13.5	0.0156	42900	429	0.0005
	Iprovalicarb	0.12	9890	989	0.0001	22700	227	0.0005
	Metrafenone	0.21	228	22.8	0.0092	820	8.2	0.0256
	Tetraconazol	0.02	300	30	0.0007	4400	44	0.0005
2	Boscalid	0.02	125	12.5	0.0016	2700	27	0.0007
	Dimethomorph	0.1	56	5.6	0.0179	6100	61	0.0016
	Folpet	4.53	8.1	0.81	**5.5926**	680	6.8	0.6662
	Fluopicolide	0.04	155	15.5	0.0026	360	3.6	0.0111
	Fluopyram	0.13	135	13.5	0.0096	42900	429	0.0003
	Iprovalicarb	3.05	9890	989	0.0031	22700	227	0.0134
	Kresoxim-methyl	0.22	13	1.3	0.1692	190	1.9	0.1158
	Metrafenone	0.05	228	22.8	0.0022	820	8.2	0.0061
	Myclobutanil	0.73	200	20	0.0365	2000	20	0.0365
	Penconazole	0.03	360	36	0.0008	1130	11.3	0.0027
	Pyrimethanil	0.03	1600	160	0.0002	10560	105.6	0.0003
	Zoxamide	0.14	4	0.4	0.3500	160	1.6	0.0875
	Dimethoate	0.06	400	40	0.0015	30200	302	0.0002
3	Folpet	0.58	8.1	0.81	0.7160	680	6.8	0.0853
	Fludioxonil	0.07	40	4	0.0175	230	2.3	0.0304
	Fluopyram	0.20	135	13.5	0.0148	42900	429	0.0005
	Dimethoate	0.02	400	40	0.0005	30200	302	0.0001
4	Boscalid	0.15	125	12.5	0.0120	2700	27	0.0056
	Folpet	0.20	8.1	0.81	0.2469	680	6.8	0.0294
	Fludioxonil	0.22	40	4	0.0550	230	2.3	0.0957
	Fluopyram	0.18	135	13.5	0.0133	42900	429	0.0004
	Iprovalicarb	0.11	9890	989	0.0001	22700	227	0.0005
	Myclobutanil	0.03	200	20	0.0015	2000	20	0.0015
	Chlorpyrifos-methyl	0.02	5	0.5	0.0400	410	4.1	0.0049
	Dimethoate	0.03	400	40	0.0008	30200	302	0.0001

5	Boscalid	0.26	125	12.5	0.0208	2700	27	0.0096
	Famoxadone	0.15	1.4	0.14	**1.0714**	11	0.11	**1.3636**
	Folpet	0.73	8.1	0.81	0.9012	680	6.8	0.1074
	Fludioxonil	0.25	40	4	0.0625	230	2.3	0.1087
	Fluopicolide	0.53	155	15.5	0.0342	360	3.6	0.1472
	Fluopyram	0.23	135	13.5	0.0170	42900	429	0.0005
	Iprovalicarb	0.36	9890	989	0.0004	22700	227	0.0016
	Metalaxyl M	0.24	9100	910	0.0003	27000	270	0.0009
	Metrafenone	0.3	228	22.8	0.0132	820	8.2	0.0366
	Myclobutanil	0.24	200	20	0.0120	2000	20	0.0120
	Tebuconazole	0.05	10	1	0.0500	4400	44	0.0011
	Zoxamide	0.02	4	0.4	0.0500	160	1.6	0.0125
	Chlorpyrifos-methyl	0.02	5	0.5	0.0400	410	4.1	0.0049
