# Supplementary material for: Pesticide exposure affects reproductive capacity of common toads (Bufo bufo) in a viticultural landscape
Source: Ecotoxicology. 2021 Jan 20;30(2):213–23. doi: 10.1007/s10646-020-02335-9 (PMC7902574; doi:10.1007/s10646-020-02335-9)
Supplement: Supplementary file 3 — Table S3 [file 10646_2020_2335_MOESM3_ESM.pdf]

Supplementary material Table S3

Pesticide exposure affects reproductive capacity of common toads (*Bufo bufo*) in a viticultural landscape

Elena Adams[1*], Christoph Leeb[1], Carsten A. Brühl[1]
[1]iES Landau, Institute for Environmental Sciences, University of Koblenz-Landau, Fortstraße 7, 76829 Landau, Germany
*Corresponding author: adams@uni-landau.de

Table S3. Results of the Kendall-Theil Sen Siegel regression model to identify whether the pesticide contamination of ponds (sum of toxic units, STU) affects the investigated reproduction endpoints.

	Coefficient	Estimate	df	*p*
Fecundity	STU	13.52	50	< 0.001
Fertilization rate	STU	-0.24	46	< 0.001
Offspring survival	STU	-3.93	46	< 0.001
Offspring fitness	STU	-0.04	48	< 0.001
