# Supplementary material for: Pesticide exposure affects reproductive capacity of common toads (Bufo bufo) in a viticultural landscape
Source: Ecotoxicology. 2021 Jan 20;30(2):213–23. doi: 10.1007/s10646-020-02335-9 (PMC7902574; doi:10.1007/s10646-020-02335-9)
Supplement: Supplementary file 4 — Table S4 [file 10646_2020_2335_MOESM4_ESM.pdf]

Supplementary material Table S4

Pesticide exposure affects reproductive capacity of common toads (*Bufo bufo*) in a viticultural landscape

Elena Adams[1*], Christoph Leeb[1], Carsten A. Brühl[1]
[1]iES Landau, Institute for Environmental Sciences, University of Koblenz-Landau, Fortstraße 7, 76829 Landau, Germany
*Corresponding author: adams@uni-landau.de

Table S4. Determined median, mean and standard deviation for the investigated reproductive endpoints and ponds.

Endpoint	Pond	Median	Mean	Standard deviation
Fecundity (eggs/g)	A	46	49	11
	B	55	54	11
	C	68	70	11
	D	71	69	23
	E	81	74	30
Fertilization rate (%)	A	99.2	98.9	0.9
	B	97.8	98.96	3.6
	C	99.7	100.0	0.5
	D	94.5	96.1	7.1
	E	92.0	94.4	8.5
Offspring survival (%)	A	97.0	98.9	4.1
	B	92.9	93.3	4.8
	C	94.7	95.0	4.2
	D	88.9	92.1	8.9
	E	54.2	66.3	36.0
Tadpole length (mm)	A	12.5	12.4	0.5
	B	11.8	11.8	0.5
	C	11.4	11.3	0.4
	D	11.8	11.7	0.8
	E	11.3	11.2	0.4
